# PredRSA: a gradient boosted regression trees approach for predicting protein solvent accessibility

**DOI:** 10.1186/s12859-015-0851-2

**Published:** 2016-01-11

**Authors:** Chao Fan, Diwei Liu, Rui Huang, Zhigang Chen, Lei Deng

**Affiliations:** School of Software, Central South University, No.22 Shaoshan South Road, Changsha, 410075 China; Shanghai Key Laboratory of Intelligent Information Processing, No.220 Handan Road, Shanghai, 200433 China

**Keywords:** Solvent accessibility, Sequence features, Gradient boosted regression trees

## Abstract

**Background:**

Protein solvent accessibility prediction is a pivotal intermediate step towards modeling protein tertiary structures directly from one-dimensional sequences. It also plays an important part in identifying protein folds and domains. Although some methods have been presented to the protein solvent accessibility prediction in recent years, the performance is far from satisfactory. In this work, we propose PredRSA, a computational method that can accurately predict relative solvent accessible surface area (RSA) of residues by exploring various local and global sequence features which have been observed to be associated with solvent accessibility. Based on these features, a novel and efficient approach, Gradient Boosted Regression Trees (GBRT), is first adopted to predict RSA.

**Results:**

Experimental results obtained from 5-fold cross-validation based on the Manesh-215 dataset show that the mean absolute error (MAE) and the Pearson correlation coefficient (PCC) of PredRSA are 9.0 % and 0.75, respectively, which are better than that of the existing methods. Moreover, we evaluate the performance of PredRSA using an independent test set of 68 proteins. Compared with the state-of-the-art approaches (SPINE-X and ASAquick), PredRSA achieves a significant improvement on the prediction quality.

**Conclusions:**

Our experimental results show that the Gradient Boosted Regression Trees algorithm and the novel feature combination are quite effective in relative solvent accessibility prediction. The proposed PredRSA method could be useful in assisting the prediction of protein structures by applying the predicted RSA as useful restraints.

## Background

Since the concept of solvent accessibility was first introduced by Lee and Richards [1], defined as the surface area of a protein that is accessible to a spherical solvent while probing the surface of that molecule, it has been considered as a key factor for understanding protein structure and function [2]. Predicting the three-dimensional (3D) structures of proteins from their one-dimensional sequences is a challenging issue because of the increasing gap between the enormous number of protein sequences and the number of known structures. Studies of solvent accessibility in proteins have provided many useful insights into the 3D structures of proteins [3]. Furthermore, knowledge of solvent accessibility has proved useful for structural domains identification [4], fold recognition [5], binding region identification [6–8] and protein intrinsic disorder [9]. The solvent accessibility is particularly important because it is associated with the spatial arrangement and packing of amino acids during the process of protein folding. It also plays an important role in predicting the active sites of protein-protein or protein-ligand binding [10].

In many earlier studies, the solvent accessibility prediction was taken as a classification problem with varying thresholds, two-state (exposed or buried) or three-state (exposed, intermediate or buried) [11–15]. However, there is no standard definition for the thresholds of solvent accessibility states. For instance, a residue may be predicted to be exposed state based on a relative solvent accessibility threshold of 10 %, but the same residue may be predicted to be buried state based on a threshold of 20 %. In view of this, it is necessary to predict the real values of solvent accessibility. Some representative machine learning techniques have been proposed to predict the real values of solvent accessibility, including multiple linear regression [16], support vector regression [17–19], neural network [20, 21], energy optimization [22] and nearest neighbor method [23].

For the real-valued solvent accessibility prediction, Ahmad et al. [20] proposed a neural network method with only single sequence information as the input features. The result showed that this method achieved a MAE of 18.0–19.5 % on different data sets. Adamczak et al. [21] employed evolutionary information in the form of position-specific scoring matrix (PSSM) profile to train a neural network-based regression for the prediction. Compared with the single sequence based neural network [20], the prediction performance was improved and the MAE decreased by about 5 % on the PFAM database [24]. Subsequently, Lee et al. [16] applied PSSM profile by constructing a correlation matrix different window positions to train a multiple linear regression method. The result showed a performance of 16.6 % MAE and 0.63 PCC on the Barton-502 dataset. Garg et al. [25] took multiple sequence alignment and secondary structure as input features to predict RSA based on a feed-forward neural network. The result indicated that a lower MAE achieved on CASP6 was 15.9 % and a higher PCC was 0.68.

Although these methods for surface accessibility prediction were developed, several issues still exist and make surface accessibility prediction a very challenging task. Mainly, there are three reasons: (1) specific biological properties for precisely predicting surface accessibility are not fully exploited, and no single parameter can definitely estimate the accessible surface area, various combinations of different feature types, including PSSM profiles, secondary structure features, native disorder features as well as other global sequence features [26], need to be investigated comprehensively; (2) the performance of the existing methods is still unsatisfactory, especially in terms of independent testing and (3) high-performance ensemble learning algorithms such as boosted regression trees haven’t been intensively used in this area.

In this article, we propose a new and efficient approach, PredRSA (Prediction of Relative Solvent Accessible surface area), that integrates gradient boosted regression trees (GBRT) algorithm with multiple sequence-based features (position-specific scoring matrix, secondary structure, conservation score, native disorder) and a global feature (side-chain environment) to predict RSA. We have benchmarked PredRSA using the Manesh training dataset and an independent dataset. Results show that PredRSA significantly outperforms the state-of-the-art methods and indicate that the GBRT algorithm and the novel feature combination are important determinants in the prediction of RSA.

## Methods

### The GBRT algorithm

Our approach utilizes an ensemble regression algorithm for predicting RSA values of amino acid residues in a protein sequence. Generally, a target residue in sequence can be described as an *n*-dimension vector. Let us denote an amino acid residue by ***x***=(*x*_1_,*x*_2_,⋯,*x*_*n*_) where *x*_*i*_∈***R*** and the corresponding real-valued RSA by *y*. The goal of predicting RSA real value of the amino acid residue in sequence is to find a function *F*^∗^(***x***) that maps ***x*** to *y*, such that over the joint distribution of all (*y*,***x***)-values, the expected value of some specified loss function *Ψ*(*y*,*F*(***x***)) is minimized as follows:

(1) $$ \begin{aligned} \ {F^{*}(\boldsymbol{x})} &= \mathop{arg\, min}\limits_{F(\boldsymbol{x})} \ {E_{y,\boldsymbol{x}}\Psi (y,F(\boldsymbol{x}))} \\ &=\mathop{arg\, min}\limits_{F(\boldsymbol{x})} \ {E_{\boldsymbol{x}}[\!E_{y}(\Psi (y,F(\boldsymbol{x}))|\boldsymbol{x}]} \end{aligned}  $$

Let $\left \{ y_{i},x_{i} \right \}_{1}^{N}$ be a set of training data, *N* is the number of all amino acid residues in the training set. The GBRT algorithm iteratively constructs M different weak learners *h*(***x***,*Θ*_1_),⋯,*h*(***x***,*Θ*_*M*_) which consist of regression trees of fixed size from training set and constructs the following additive function *F*(***x***):

(2) $$ F(\boldsymbol{x})=\beta_{0}+\sum_{m=1}^{M}h(\boldsymbol{x},\Theta_{m})  $$

where *β*_*m*_ and *Θ*_*m*_ are a weight and vector of parameters for the *m*th weak regression tree *h*(***x***,*Θ*_*m*_), respectively, and *β*_0_ is an initial value. Both the weight *β*_*m*_ and the parameters *Θ*_*m*_ are iteratively determined from *m*=1 to *m*=*M* so that a loss function *Ψ*(*y*,*F*(***x***)) is minimized. That is, *β*_*m*_ and *Θ*_*m*_ for the *m*th regression tree are determined as follows:

(3) $$\begin{array}{@{}rcl@{}} (\beta_{m},\Theta_{m})= \mathop{arg\, min}\limits_{\beta,\Theta} \ {\sum_{i=1}^{N}\Psi (y_{i},F_{m-1}(\boldsymbol{x}_{i})+\beta h(\boldsymbol{x}_{i},\Theta))} \end{array} $$

where *F*_0_(***x***) is an initial value and given by $F_{0}(\boldsymbol {x})=\beta _{0}= \mathop {arg\, min}_{\beta } \ {\sum _{i=1}^{N}\Psi (y_{i},\beta)}$, *F*_*m*−1_(***x***) is the (*m*−1)th additive function combined from the first to the (*m*−1)th weak regression tree.

However, in general, it is not straightforward to solve Eq. (3). Therefore, GBRT separately and approximately estimates (*β*_*m*_,*Θ*_*m*_) with a simple two-step fashion [27]. For the estimation of the parameters *Θ*_*m*_, we determine them so that the function defined by the regression tree approximates a gradient with respect to the current function *F*_*m*−1_(***x***) in the sense of least-square error as follows:

(4) $$ \Theta_{m}= \mathop{arg\, min}\limits_{\Theta} \ {\sum_{i=1}^{N}(\tilde{y}_{im}-h(\boldsymbol{x}_{i},\Theta))^{2}}  $$

where $\tilde {y}_{\textit {im}}$ is the gradient and is given by

(5) $$ \tilde{y}_{im}=-\left[\frac{\partial \Psi (y_{i},F(\boldsymbol{x}_{i}))}{\partial F(\boldsymbol{x}_{i})}\right]_{F(\boldsymbol{x})=F_{m-1}(\boldsymbol{x})}  $$

When the *m*th regression tree using the *Θ*_*m*_ has *L*_*m*_ leaf nodes, the regression tree is given by

(6) $$ h\!\left(\boldsymbol{x},\left\{R_{lm} \right\}_{l=1}^{L_{m}}\right)=\sum_{l=1}^{L_{m}}\bar{y}_{lm}l(\boldsymbol{x}\in R_{lm})  $$

where *R*_*lm*_ is a disjoint region that the *l*th leaf node of the *m*th regression tree defines. *l*(.) is a Boolean function that outputs 1 in case the argument of the function is true. $\bar {y}_{\textit {lm}}$ is a constant for the *R*_*lm*_th region, defined as the mean of training data that belongs to the *l*th leaf node of the *m*th regression tree. The weight *β*_*m*_ can be straightforwardly chosen using line search:

(7) $$\begin{array}{@{}rcl@{}}  \beta_{m}= \mathop{arg\, min}\limits_{\beta} \ {\sum_{i=1}^{N}\Psi\! \left(\!y_{i},F_{m-1}\left(\boldsymbol{x}_{i}\right)\,-\,\beta \frac{\partial \Psi\left(y_{_{i},F_{m-1}\left(\boldsymbol{x}_{i}\right)}\right)}{\partial F_{m-1}(\boldsymbol{x}_{i})}\!\right)} \!\!\!\end{array} $$

Then, a new additive function *F*_*m*_(***x***) is updated as follows:

(8) $$ F_{m}(\boldsymbol{x})=F_{m-1}(\boldsymbol{x})+\nu \sum_{l=1}^{L_{m}}\beta_{m}\bar{y}_{lm}l(\boldsymbol{x}\in R_{lm})  $$

where 0<*ν*<1 is a shrinkage parameter, also called the learning rate to scale the step length the the gradient descent procedure. In this work, we take Huber loss function [28] as the loss function given by

(9) $$ \Psi (y,F) = \begin{cases} \frac{1}{2}(y-F)^{2}\ \ if\ |y-F|\leqslant \delta \\ \delta (|y-F|-\delta /2)\ \ if \ |y-F|> \delta \end{cases}  $$

Hence, in Eq. (5), $\tilde {y}_{\textit {im}}$ becomes:

(10) $$\begin{array}{@{}rcl@{}} {}\tilde{y}_{im} = \begin{cases} y_{i}-F_{m-1}(\boldsymbol{x}_{i})\ \ if\ |y_{i}-F_{m-1}(\boldsymbol{x}_{i})|\leqslant \delta \\ \delta.sign(y_{i}-F_{m-1}(\boldsymbol{x}_{i}))\ \ if \ |y_{i}-F_{m-1}(\boldsymbol{x}_{i})|> \delta \end{cases} \end{array} $$

The value of the transition point *δ* depends on the iteration number *m*.

Finally, the resulting RSA value *y* corresponding to the amino acid residue ***x*** is given by: *y*=*F*_*M*_(***x***).

### Sequence encoding schemes

Selecting appropriate features is a crucial step because it directly determines the prediction performance. In this article, we explore various sequenced-based features which have been shown to be related to the solvent accessibility or ever applied in the similar issues. These features include PSSM profiles [29–31], PSIPRED-predicted secondary structure [32], DISOPRED-predicted native disorder [33], conservation score and side-chain environment compositions [34]. In this section, a more detail description about how to extract and encode these different sequence-based features as follows.

#### PSI-BLAST-based profiles

Position-specific scoring matrix (PSSM) of a residue which is achieved by the PSI-BLAST program contains important evolutionary information that determines whether this residue is conserved in its family of related proteins. Each element in the PSSM represents the probability of each residue position in the multiple sequence alignment. Plenty of previous studies have shown that multiple sequence alignments in the form of PSSM can substantially improve overall prediction performance [35–38]. In this article, the PSSM profile for each protein sequence is generated with default parameters (3 iterations and 0.001 of E-value cutoff) against the non-redundant (nr) dataset obtained from the NCBI. We encode each residue using a local sliding window approach based on the PSSM profiles. The PSSM profile generated by PSI-BLAST consists of the likelihood of a particular residue substitution at a specific position. These likelihood values are normalized to [0,1] by standard logistic function:

(11) $$ {x}'=\frac{1}{1+exp\left (-x \right)}  $$

where *x* is the score derived from the PSSM profile and *x*^′^ is the standardized value of *x*. For a given residue, its local sequence fragment is extracted and encoded as a 20×(2*l*+1)-dimensional vector by using a sliding window scheme where *l* denotes the half window size and *L*=2*l*+1 is the whole window length. Furthermore, the predictive performance of a variety of different local window sizes L (from 3–17) has been evaluated to select the optimal local window size L for the RSA prediction. Finally, in this encoding scheme, a residue is encoded by a 20×*L*=20×(2*l*+1)-dimensional vector.

In addition, we try to introduce residue conservation score for the solvent accessibility prediction. The value of sequence conservation for residue is a measure of how often a given residue is seen at an equivalent position in an equivalent protein across different species. Generally, residue conservation score is proportional to its buried degree. The conservation score is obtained by PSI-BLAST search as well [39, 40].

#### PSIPRED-predicted secondary structure information

In this work, we use the PSIPRED program to predict the secondary structure information. PSIPRED provides highly accurate prediction for protein secondary structures by applying a feed-forward neural network. The outputs of PSIPRED are encoded by the probability profiles of three secondary structures (C for coil, H for helix and E for strand). Some previous works have shown that incorporation of PSIPRED-predicted secondary structure information can significantly improve the prediction performance [25, 41].

Analogously, for a given residue, its three-state secondary structure profiles are extracted and encoded using a sliding window of *L*=2*l*+1 consecutive residues. Therefore, in this encoding scheme, a residue is composed of a 3×*L*=3×(2*l*+1)-dimensional vector.

#### DISOPRED-predicted native disorder information

In the past decade, protein disorder or unstructured regions have received considerable attention in that they are commonly responsible for important protein function. As such, there has been an increasing interest in studying such regions in proteins. Unstructured regions are found to be associated with molecular assembly, protein modification and molecular recognition [42–44]. Research shows unstructured regions have a large solvent accessible area, which explains why polar and charged residues which favorably interact with water are prevalent in these regions [45]. The conclusion is that disordered regions are strongly correlated with local solvent accessibility areas. Local solvent accessibility values are often used to find the disordered regions as well [46, 47].

In order to further improve the performance, in this study, we use DISOPRED program to output the predicted possibility of each residue being natively disordered or ordered. Similarly, a residue is encoded by a 2×*L*=2×(2*l*+1)-dimensional vector in this encoding scheme.

#### Side-chain environment

The concept of side-chain environment was first purposed by Eisenberg et al. [34] and used to identify protein sequences that fold into a known three-dimensional structure. Then Li et al. [39] utilized it for prediction of protein-protein binding site.

**Fig. 1:**
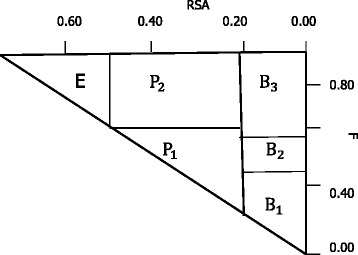
The definition of the six side-chain environment categories. This figure shows the classification method of side-chain environment. *RSA* represents the relative accessible surface areas and *F* represents the fraction of the whole side-chain area covered by polar atoms. If *R* *S* *A*<0.09, the residue will be placed into class B(buried). If 0.09≤*R* *S* *A*<0.36, the residue will be placed into class P(partial buried). If $RSA\geqslant 0.36$, the residue will be placed into class E(exposed). Within class B, if *F*<0.45, the residue will be placed into *B*_1_, if 0.45≤*F*<0.58, the residue will be placed into *B*_2_, and if $F\geqslant 0.58$, the residue will be divided into class *B*_3_. In class P, if *F*<0.67, the reside will be divided into class *P*_1_, and if $F\geqslant 0.67$, the reside will placed into class *P*_2_

The side-chain environment of a residue is typically defined as buried, partially buried, or exposed based on its solvent accessible surface area. The buried and partially buried residue environments can be further subdivided according to the fraction of side-chain area covered by polar atoms. Based on this, we divide the side-chain environment of a residue into six classes (see Fig. 1). The detailed definition of the side-chain environment were described in the work of Eisenberg et al. [34].

### Framework of PredRSA

**Fig. 2:**
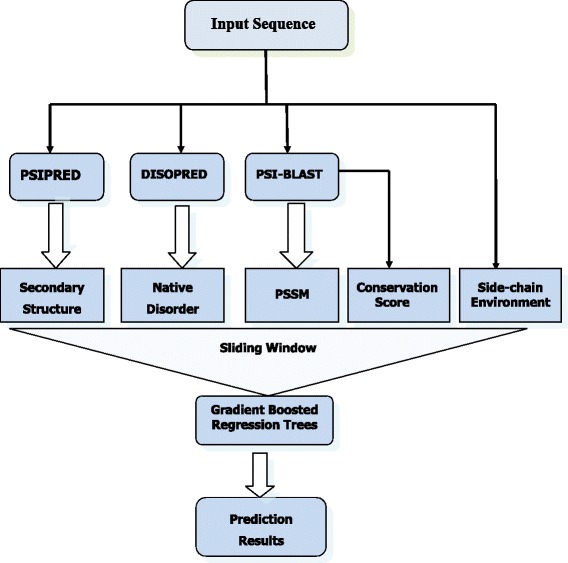
The framework of PredRSA for protein relative solvent accessibility prediction. Five different types of sequence-derived features are generated and used as input to build the GBRT model. These features consist of PSSM in the form of PSI-BLAST profiles, predicted secondary structure information by PSIPRED, predicted native disorder information by DISOPRED, conservation score and side-chain environment

In this subsection, we describe the PredRSA framework that uses an accurate and effective ensemble computational approach for real values of relative solvent accessibility prediction from protein primary sequences. We are interested in investigating the influence of various sequence-based features and their combinations on the prediction performance of solvent accessibility. In order to fully exploit the sequence-derived features for RSA prediction, we propose a novel PredRSA approach which incorporates five different types of sequence-derived features as inputs. They are four local features (position-specific scoring matrix, secondary structure, conservation score, native disorder) and a global feature (side-chain environment). Figure 2 illustrates the flowchart of our proposed approach.

To determine the optimal local sliding window size *L* and the iterative tree number *M*, we calculate the prediction performance for *L* in the range of 3–17 with a step of 2 and *M* in the range of 100–1500 with a step of 50 using a grid search method. With *L*=7 and *M*=800, the PredRSA approach achieves the best performance for the RSA prediction.

## Results and discussion

### Datasets

Two non-homologous datasets of proteins chains with pair-wise sequence similarity less than 25 % have been used in order to objectively compare our approach with other available methods developed previously. One dataset is consisted of 215 proteins, which was also used earlier by Manesh et al. [11] for solvent-accessible surface area of residues prediction. The other dataset is consisted of 502 proteins, obtained from the Cuff and Barton [48] dataset of 513 proteins, selected by removing those sequences, which have less than 30 residues. These two datasets have been referred to as Manesh-215 and CB-502, respectively. However, since the Manesh-215 dataset was widely used by researchers to benchmark prediction methods [18, 20, 25, 49], taking into account comparative purposes, we use Manesh-215 as the main data set for evaluation and analysis.

To further evaluate the performance of existing methods and the method developed in the present study, we also generate an independent dataset of CASP10 proteins. Originally, it contains 85 proteins [50], and we have removed 17 structures (containing chains) by using PISCES culling sever [51] with 25 % sequence similarity cutoff including X-ray (less than 3.0 Å resolution and 0.3 of R-factor) and NMR structures which contain more than 50 residues. Finally, the remaining 68 proteins are used for independent test.

### Calculation of RSA

In this work, we take relative solvent accessibility, also called relative solvent accessible surface area (RSA) as the prediction of solvent accessibility. The RSA of a residue in a protein chain is a normalized value from 0–1. It is calculated as the ratio by dividing the solvent accessible surface area (ASA) by the maximum solvent accessibility according to Manesh’s work [11] which uses Gly-X-Gly extended tripeptides. The values of ASA are calculated using DSSP [52] for all considered protein structures.

### Evaluation measures

To measure the performance of real-valued RSA predictions, three widely used measures for real value RSA prediction are adopted in this study.

The first measure, mean absolute error (MAE), is defined as the average difference between the predicted and experimental RSA values of all residues:

(12) $$ MAE=\frac{\sum \left | {RSA}_{predicted} -{RSA}_{experimental}\right |}{N}  $$

The second measure is the root mean square error (RMSE), which is defined as follows:

(13) $$ RMSE=\sqrt{\frac{1}{N}\sum_{N}^{i=1}\left ({RSA}_{predicted} -{RSA}_{experimental}\right)^{2}}  $$

The third measure, Pearson correlation coefficient (PCC), the ratio of the covariance between the predicted and experimental RSA values which is given by:

(14) $$ PCC=\frac{\sum_{i=1}^{N}(x_{i}-\bar{x})(y_{i}-\bar{y})}{\sqrt{\sum_{i=1}^{N}(x_{i}-\bar{x})^{2}\sum_{i=1}^{N}(y_{i}-\bar{y})^{2}}}  $$

where *N* is the total number of residues in a protein sequence to predict; *x*_*i*_ and *y*_*i*_ are the experimental and predicted RSA values of the *i*-th residue, respectively; $\bar {x}$ and $\bar {y}$ are their corresponding means. *PCC*=1 indicates that the two sets of values are fully correlated, while *PCC*=0 indicates that they are completely uncorrelated.

Two-state (buried or exposed) predictions are evaluated according to various thresholds of RSA. Prediction accuracy which is defined by the percentage of correctly predicted residues divided by the total number of residues and Matthews correlation coefficient (MCC) are given as follows:

(15) $$\begin{array}{*{20}l} ACC=\frac{N_{b}+N_{e}}{N} \end{array} $$

(16) $$  MCC=\frac{TP\times TN-FP\times FN}{\sqrt{(TP+FP)(TP+FN)(TN+FP)(TN+FN)}}  $$

where *N* is the total number of residues in a chain, *N*_*b*_ and *N*_*e*_ represent the number of residues correctly predicted as buried and exposed, respectively. *TP*,*TN*,*FP* and *FN* are the numbers of the true positives, true negatives, false positives and false negatives, respectively.

### Effect of different sequence encoding schemes on the prediction performance

**Fig. 3:**
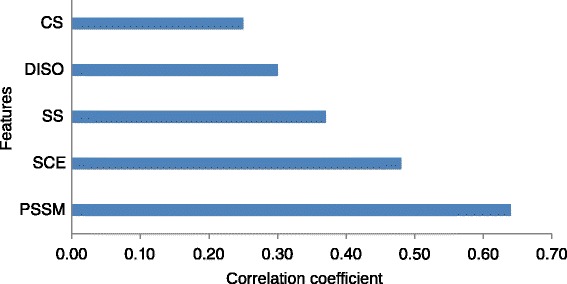
The importance of the five relevant features used in PredRSA. PSSM, SS, DISO, SCE and CS stand for position specific scoring matrix, protein secondary structure, protein native disorder, side-chain environment and conservation score, respectively

We analyze the importance or contribution for each individual feature, which is useful to identify those features that have the most significant influence on overall prediction performance. The performance of each individual predictive is shown in Fig. 3. The feature of side-chain environment is first introduced to predict RSA and it is strongly related to solvent-accessible surface areas.

Table 1 compares the prediction performance of five different combinations of sequence-based features on Manesh-215 with 5-fold cross-validation. As shown in Table 1, the prediction performance of combining all five types of features is the best. It suggests that comprehensive sequence encoding schemes can improve the predictive performance. More importantly, incorporating side-chain environment into the model can significantly increase the prediction performance.

**Table 1:** Prediction of real-valued RSA using the GBRT algorithm based on five different sequence encoding schemes that incorporate various combinations of sequence features

Feature	RMSE(%)	MAE(%)	PCC
PSSM	13.87	10.26	0.67
PSSM+DISO	13.38	9.93	0.69
PSSM+DISO+SS	13.04	9.64	0.71
PSSM+DISO+SS+SCE	12.23	8.99	0.74
PSSM+DISO+SS+SCE+CS	12.07	8.86	0.75

### Performance comparison with other regression approaches

In this section, we compare the performance of PredRSA with that of other five existing real value RSA predictors, including a quadratic programming and buriability energy function for solvent accessibility prediction (QBES) [22], a neural network-based method using multiple sequence alignment and secondary structure (SARpred) [25], an improved two-layer neural network (Real-SPINE) [53], a support vector regression using enhanced PSSM features (SVR) [54] and an ensemble of artificial neural networks method (NetSurfP) [55]. Table 2 summarizes the results of these methods. We observe that our method achieves a significantly better performance over the compared predictors. Particularly, the PCC value of PredRSA is approximately 5 % higher than that of the previous predictors on Manesh-215. It is worth to point out that experimental maximum solvent accessibility scores are varied based on different references [11, 56, 57]. A higher maximum solvent accessibility score will lead to a lower RSA value, and thus a relatively lower MAE is obtained according to the definition of MAE. One reason for the differences of MAE between PredRSA and the other methods is that these methods may use different maximum solvent accessibility scores. On the other hand, the prediction precision of PredRSA is higher than that of the other methods and yields a lower MAE.

**Table 2:** Performances comparison in predicting real values: PredRSA vs. other existing methods

	Manesh-215		CB-502	
Method	MAE(%)	PCC	MAE(%)	PCC
QBES	-	0.52	-	0.49
SARpred	14.9	0.68	15.9	0.66
SVR	14.2	0.69	14.8	0.68
Real-SPINE	13.8	0.70	14.5	0.68
NetSurfP	13.6	0.70	14.3	0.71
PredRSA	9.0	0.75	9.4	0.73

### Performance comparison for two-state prediction

In the past, a plenty of approaches have been proposed for predicting the states (exposed or buried) of residues. Here we examine the performance of our method in terms of two-state prediction. We assign the label of a residue based on its predicted RSA value and a chosen threshold. Table 3 shows the performance of the two-state classification prediction.

**Table 3:** Prediction performance of two-state classification based on different thresholds

	Manesh-215		CB-502		CASP10	
Threshold(%)	ACC(%)	MCC	ACC(%)	MCC	ACC(%)	MCC
5	80.1	0.54	77.9	0.50	78.5	0.48
10	81.7	0.63	79.0	0.58	79.1	0.57
20	81.0	0.61	80.5	0.60	78.3	0.56
25	81.2	0.58	81.0	0.57	79.7	0.56
30	82.4	0.54	82.1	0.52	80.5	0.51
40	87.1	0.42	86.8	0.39	85.0	0.40
50	93.2	0.25	93.0	0.23	91.2	0.30

We also compare the classification accuracy of PredRSA with that of other approaches by different thresholds. The threshold is used to determine the state (exposed or buried) of a predicted real value. For example, a 5 % threshold means a residue is defined as buried if its RSA value is less than 5 %. The methods for comparison include SARpred [25], pace regression algorithm (PR) [58], two-stage SVR [19] and SVR [54]. The prediction accuracy is showed in Table 4. Our method yields more than 80 % classification accuracy at any thresholds and obtains almost the highest accuracy across all the thresholds.

**Table 4:** Performance comparison of two-state classification: PredRSA vs. other existing predictors

	Accuracy for two-states prediction(%)						
Method	5 %	10 %	20 %	25 %	30 %	40 %	50 %
SARpred	74.9	77.2	77.7	-	77.8	78.1	80.5
PR	76.8	74.8	75.3	76.7	77.7	79.8	86.3
SVR	80.9	80.1	78.7	-	-	-	80.8
Two-stageSVR	81.1	78.7	77.6	77.3	-	-	79.5
PredRSA	80.0	81.6	80.9	81.1	82.2	87.1	93.2

### Independent test on the CASP10 dataset

An independent test (CASP10) is constructed to further validate the usability of our PredRSA method. We train the classifiers based on the Manesh-215 dataset and test against the CASP10 dataset which contains 68 proteins. Other state-of-the-art methods including SPINE-X [59] and ASAquick [60] are also evaluated. SPINE-X uses a multistep neural-network algorithm by coupling secondary structure prediction with prediction of solvent accessibility and backbone torsion angles in an iterative manner, while ASAquick utlizes solely sequential widow information and global features with a general neural network method. The Pearson correlation coefficient of PredRSA is 0.71, which outperform the results of SPINE-X and ASAquick by a rate of 2 % (0.69) and 4 % (0.67).

### Case study

**Fig. 4:**
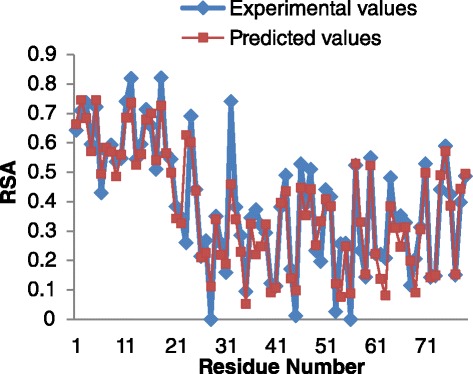
Predicted and experimental values (%) of RSA for each residue of CASP10 T0675

For a better understanding of the power of our proposed PredRSA approach and illustrating the significance of PCC, RMSE and MAE measures used in this work, an example of the real-valued RSA for T0675 (Insulinoma-associated protein) from CASP10 is shown in Fig. 4. For this protein, our method gives a MAE of 5.31 %, a RMSE of 7.91 % and a PCC of 0.92. From Fig. 4, we can see that the majority of its predicted RSA values are in good agreement with the corresponding experimental RSA values calculated by DSSP, except for several separate positions.

**Fig. 5:**
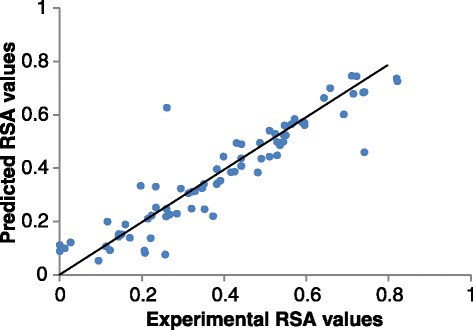
Correlation between experimental RSA values and predicted RSA values of CASP10 T0675. The Pearson correlation coefficient score is 0.92 and the most buried residues are well predicted with the RSA values near zero

In Fig. 5, the continuous real-value prediction of RSA and the actual continuous values are shown. Significant correlation between the true values and the predicted values is obtained.

### Residue-specific variation in prediction error

**Fig. 6:**
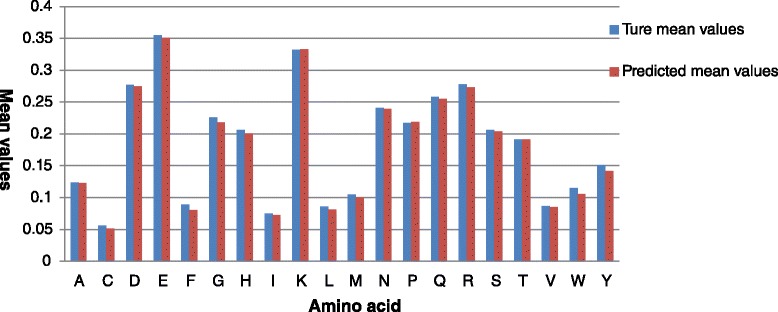
Comparison between true mean values and predicted mean values for 20 amino acids on the Manesh-215 dataset

In order to assess the prediction performance of various types of residues, we further calculate the average RSA values on the Manesh-215 dataset for all 20 amino acids (Fig. 6) from the PredRSA predictor. As can be seen from Fig. 6, an overwhelming majority of types of amino acids are predicted with <1 % mean error. All types of amino acids are predicted with <2 % mean error in our method. In particularly, we find that the true mean RSA values are in highly accord with the predicted mean RSA values for these amino acids, such as A (Ala), K (Lys), N (Asn), T (Thr).

**Fig. 7:**
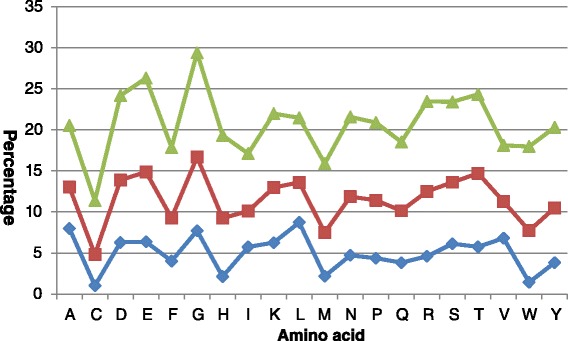
Mean predicted errors of 20 amino acids on the Manesh-215 dataset. The green line represents standard root mean square error, the red line represents mean absolute error and the blue line represents the corresponding data distribution of 20 amino acids

Furthermore, we calculate the prediction errors of 20 amino acids on the Manesh-215 dataset. Figure 7 shows the mean absolute error (MAE) and the standard root mean square error (RMSE) of 20 amino acids. As expected, G (Gly) shows the highest MAE and RMSE due to its flexibility, and other polar residues show similar behavior. Hydrophobic amino acids including C (Cys), F (Phe), M (Met) and W (Trp) are better predicted than less hydrophobic amino acids. These results are also in good agreement with our PredRSA method.

## Conclusions

Knowledge of residue solvent accessibility gives useful insights into protein structure and function prediction. In this work, we have presented PredRSA to predict real-valued relative solvent accessibility as well as classification state (buried or exposed) of a target residue. The method is based on a gradient boosted regression trees (GBRT) algorithm combined with a novel set of features. The 5-fold cross-validated correlation coefficient between predicted and experimental RSA (0.75) is significantly better than existing methods on the Manesh-215 dataset. We also performed additional independent benchmark tests of PredRSA on the CASP10 set containing 68 proteins where we find that the proposed method outperforms existing methods. Furthermore, for prediction of discrete state, our method is able to achieve an accuracy of 79.7 % with an MCC value of 0.56 using two states classifications at a threshold of 25 %, which defines an approximately balanced division into the two classes.

Experimental results show GBRT is an efficient machine learning approach for continuous values of the solvent accessibility of a target residue. Compared with other traditional techniques, GBRT has several obvious advantages such as high prediction accuracy and stronger generalization capability.

On the other hand, PredRSA utilizes a variety of multiple sequence-derived features, including the position-specific scoring matrices and conservation score in the form of PSI-BLAST profiles, predicted secondary structure, predicted natively disordered region and side-chain environment. We have comprehensively assessed the effects of different sequence encoding schemes on the prediction performance of RSA, and the results show the prediction performance of RSA outperforms previous methods. Our work provides a complementary and useful approach towards the more accurate prediction of protein solvent accessibility.
